# AI in business operations: driving urban growth and societal sustainability

**DOI:** 10.3389/frai.2025.1568210

**Published:** 2025-03-24

**Authors:** Sharareh Shahidi Hamedani, Sarfraz Aslam, Shervin Shahidi Hamedani

**Affiliations:** ^1^Faculty of Business, UNITAR International University, Petaling Jaya, Malaysia; ^2^Faculty of Education and Humanities, UNITAR International University, Petaling Jaya, Malaysia; ^3^Department of Environmental Sciences and Engineering, NOVA School of Science and Technology, Universidade NOVA de Lisboa, Lisbon, Portugal

**Keywords:** artificial intelligence, sustainability, business operations, societal dynamics, urban growth

## 1 Introduction

Approximately 30% of smart city applications will use artificial intelligence (AI) by the end of 2025, thereby radically altering the urban sustainability landscape in the future (Yan et al., 2023). The advent of AI in reshaping traditional businesses into sustainable operations is evident. Whenever AI is brought to the forefront, it is considered a cornerstone in the business domain, enabling a transition toward more innovative and sustainable practices (Appio et al., 2024). Incorporating AI into business practices has many facets. According to Grand View Research (2023), the global AI market size was anticipated at USD 196.63 billion in 2023 and is expected to grow at a CAGR of 36.6% from 2024 to 2030. The recent fanfare surrounding AI has elevated it to a key enabler of sustainable development, prompting many companies to prioritize and integrate it into their business operations; hence, there is a stark difference between traditional and new practices. In tandem with this evolution, urban growth and societal dynamics are experiencing profound changes as AI-driven solutions come to the fore in various aspects of modern society (Shahidi Hamedani et al., 2024).

AI applications in city government, transforming conventional cities into efficient ones (Ortega-Fernández et al., 2020), have significantly shifted from functional systems to more sustainable and intelligent ones. Furthermore, from another perspective, the role of AI in optimizing business processes has surpassed comparison with its implication for improving logistics operational capabilities and reducing environmental impacts (Jorzik et al., 2024) till manufacturing reduces downtime, all of which contribute to the growth of urban economics. In the meantime, with the speedy pace of adoption of AI in business operations, it is also imperative to amalgamate with sustainable practices. Acting on this matter requires a thoughtful approach that aligns AI with social, economic, and environmental sustainability.

## 2 AI in business operations

The intersection of AI role and business operations has recently gained widespread attention. Some studies (Chen et al., 2024; Shahzadi et al., 2024) focused on AI's role in supply chain management, highlighting its role in minimizing inefficiencies and improving logistics by utilizing AI more often; supply chains become leaner and reduced carbon footprints, paving the path to sustainable operations. It is estimated that by 2026, 60% of businesses will adopt AI-powered warehouse solutions instead of just 10% in 2020 (MHI, 2024). In line with this shift (Dilmegani and Ermut, 2025), note that businesses also invest heavily in warehouse robots to enhance their supply chain management through AI technology. Robots can manage operations more efficiently and accurately by automating picking, packing, sorting, and inventory management, thus saving labor costs and accelerating order processing. Amazon, for instance, has deployed more than 200,000 robots in its warehouses to optimize operations.

AI can be used to optimize resource utilization, automate processes for improved efficiency, and enable real-time monitoring that aligns with sustainability goals (Waltersmann et al., 2021). As sustainable supply chain management focuses on reducing waste and enhancing traceability, AI-driven technologies such as machine learning and big data analytics have been pivotal in achieving these goals (Tsolakis et al., 2023).

Companies like eBay leverage AI for machine translation, enhancing decision-making and operational efficiency. Similarly, Vodafone employs AI-driven analytics to personalize services, exemplifying its transformative impact (Jorzik et al., 2024). These technologies help reduce forecasting errors, minimize excess inventory, and lower energy consumption (Sharma et al., 2020). Likewise, Smart grid protection sensors can detect defects up to 80% more accurately than traditional sensors, reducing losses and improving the system's reliability by adjusting to grid conditions dynamically (Mahadik et al., 2025). These applications contribute to urban economic growth by fostering technological innovation. AI leverages advanced techniques like deep reinforcement learning (DRL) to optimize dynamic business operations (Shuford, 2024). DRL improves supply chain management through adaptive routing and inventory optimization, dynamically adjusting to real-time changes in demand and logistics; with the help of DRL, researchers can develop systems that can dynamically adapt to changes, optimize resource utilization, and facilitate multi-objective decision-making for instance (Dehaybe et al., 2024).

In addition, it enables businesses to prevent equipment failures and minimize downtime, thereby streamlining workflows significantly (Mohan et al., 2021). Moreover, in urban centers, these advancements catalyze economic growth and foster innovation. In other words, a key contribution of AI is to facilitate smart urban development and efficient resource allocation, thereby ensuring that cities are resilient and economically prosperous (Li et al., 2024). In developing smart cities, AI has a transformative impact on urbanization trends. Through the application of AI, urban infrastructure can be optimized by improving energy efficiency, streamlining transportation, and managing housing needs; AI makes it possible to reduce traffic congestion and advance mobility in transportation systems, such as prescriptive traffic management and autonomous vehicles (Regona et al., 2024).

In cities like Singapore, AI manages real-time traffic and monitors energy consumption, setting urban efficiency benchmarks (Padhiary et al., 2025). On a similar note, Tennet TSO, a German transmission system operator, has been utilizing AI-based forecasting and IBM Watson's cognitive computing platform to anticipate renewable energy generation in real time, allowing real-time grid adjustments and maximizing clean energy use (Mahadik et al., 2025).

## 3 Sustainability and AI-driven business practices

Nowadays, sustainability is a debatable topic, and the role of AI in sustainability is inevitable. Reducing waste and environmental food print, optimizing resource utilization, and fostering a circular economy is the sprout of AI role which assists in a sustainable environment (Onyeaka et al., 2023); for example, in the agriculture industry, enhancing operational automation, a prediction model for the total agricultural output value (Sachithra and Subhashini, 2023), improving yields while reducing environmental impact. Moreover, this is apparent regarding the implications of AI and IoT in agriculture due to their ability to improve efficiency and sustainability. Agriculture leads the way with 35% of these technologies, followed by precision farming and irrigation monitoring at 16% each. Farming practices are becoming smarter and more sustainable due to these innovations, which increase yields, reduce waste, and conserve resources (Market.Us, 2024).

Similarly, smart grid technologies optimize energy distribution, lowering carbon footprints (Bhattacharya et al., 2022). As manufacturing and logistics become increasingly automated, energy consumption and operational inefficiencies will be minimized and aligned with global sustainability goals (Garrido et al., 2024).

By placing AI at the heart of sustainability, industries can grow while solving environmental and social issues. Moreover, businesses shift from traditional linear operations to circular, innovative, and efficient models (Pathan et al., 2023). The paradigm shift of AI contributes to sustainability from various aspects; for site surveying and progress monitoring, AI power drones are used to enhance decision-making, reduce energy consumption and minimize waste, and facilitate green finance in the agriculture sector and its application in the cultivation and harvesting phases (Fuentes-Peñailillo et al., 2024). While AI is crucial in ensuring sustainable business operations, implementing it brings several challenges, including ethical and privacy concerns (Fan et al., 2023).

In urban planning and infrastructure, there are also notable examples; by using data and knowledge acquired by AI, cities can shift to another level and have the potential to revolutionize city development, which will enable over 30% of smart city applications by 2025, including urban transportation solutions, significantly enhancing urban sustainability, social welfare, and vitality (Herath and Mittal, 2022). Furthermore, AI-enabled robots are deployed in the hospitality sector to provide personalized services and facilitate seamless guest experiences (Szpilko et al., 2023). Similarly, in the healthcare industry, AI can detect and predict diseases rapidly and accurately (Rashid and Kausik, 2024). For instance, the PRAIM study in Germany assessed AI-supported mammography screening vs. standard double reading. Out of 463,094 women screened, 260,739 were assisted by AI. With AI-supported screening, 6.7 cancers were detected out of 1,000, which is 17.6% higher than in standard screening (Eisemann et al., 2025).

## 4 Societal implications

Policies are needed to protect individual privacy in urban settings and solve concerns (Dong and Liu, 2023). AI technologies rapidly gain momentum in various industries but present challenges, including significant data security and privacy concerns. Data privacy and security protection are becoming an urgent concern (Saura et al., 2022). Acknowledging that AI adoption will have significant societal consequences, particularly when shifting employment patterns and consumer behaviors, is important (Yu et al., 2023). The rise of automation has displaced traditional jobs and created a demand for AI-specialized workers (Betts et al., 2024).

AI's role in personalizing consumer experiences underscores the ethical responsibility to protect data privacy and mitigate algorithmic biases, maintaining public trust and equity. Governments and businesses must work together to implement reskilling programs to seamlessly transition to an AI-driven world. AI's Ethical concerns, like data privacy and the digital divide, underscore the need for transparent and inclusive AI solutions (Bouhouita-Guermech et al., 2023). These challenges are amplified in urban areas, where disparities in digital access can marginalize vulnerable populations. These issues can be solved only by collaborative efforts to design AI systems prioritizing societal wellbeing and inclusiveness.

## 5 Challenges and limitations

Several challenges exist, including data integration issues, AI literacy issues, resistance to technological change, data availability, and reliance on data (Uwaoma et al., 2024). In many industries, getting clean and actionable data is time-consuming and costly. As a result, AI models cannot produce satisfactory results without robust data, undermining their potential for sustainability. Moreover, AI adoption is complicated by ethical issues (Bouhouita-Guermech et al., 2023). Ensuring equal access to technology and data privacy must be addressed so that AI benefits all sectors of society. Additionally, fostering AI literacy within organizations is of utmost importance. Many organizations resist to change due to a lack of understanding, making it difficult for them to adopt AI-driven sustainability practices in the future.

Moreover, lack of data (availability and quality) also remains a hurdle for implementing sustainability in business operations; in other words, accessing clean data is also opaque (Jorzik et al., 2024); for instance, for training DRL's models, quality datasets are critical, and data within several sustainability contexts is both sparse and expensive to collect (Equihua et al., 2024). On the other hand, the reliability of data is also another concern; according to Choudhuri (2023), 30 % of sustainability data is unreliable or has poor quality; having said that, incomplete data can fail any method of analysis and affect the decision-making process in other words without data—especially high-quality data—sustainable development is doomed to falter. A further concern is ensuring equitable access to AI since marginalized communities often face barriers to taking advantage of these developments (Kasun et al., 2024). The challenges highlighted here highlight the need for a balanced approach to AI deployment.

Without AI, the prospects of adopting sustainable business practices are becoming increasingly bleak. However, Sustainable business demands the involvement of the government and the public sector. Governments must establish policies and regulations to promote transparency and collaboration to ensure high-quality data transfer to the private sector. Policies of this kind can foster cooperation between industries, facilitating the use of AI technologies responsibly and efficiently while addressing broader sustainability goals.

The advancement of AI, however, is hindered by several limitations, including an unwillingness to change, ethical privacy concerns, and the difficulty of integrating new technology into pre-existing HR systems (Madanchian and Taherdoost, 2025). In addition, AI advancements are hampered by algorithms without common sense that cannot interpret data properly, resulting in flawed decisions (Nishant et al., 2024). As a result, clinicians' decision-making can be negatively impacted (Dratsch et al., 2023); for example, when prescribing antidepressants, clinicians were less accurate when following incorrect AI recommendations compared to a baseline or correct advice condition (Jacobs et al., 2021). The high cost of implementing AI in resource-intensive settings makes it difficult to reach a broad audience (Sommer et al., 2023). Additionally, organizational resistance to change creates a significant barrier to adopting AI in HRM since employees are reluctant to adopt AI due to concerns about data security, privacy, and possible job losses (Hassan et al., 2024).

## 6 Summary

Businesses and industries are witnessing the impact of AI as a key driver of growth, which profoundly impacts businesses in various sectors. For instance, In the context of urban development, it can be implemented to improve traffic management, infrastructure, and public transportation scheduling in a way that contributes to more livable and sustainable urban development. AI can provide businesses with the means to optimize resources, reduce inefficiencies, and embrace innovative practices, enabling them to tackle urgent environmental and economic concerns. The full benefits of AI can only be realized if businesses align their operations with clearly defined sustainability targets. Achieving this requires a strategic approach to AI, not just a technical tool for generating short-term benefits. Policymakers must develop a reliable model that fairly and equitably fosters the use of AI in a broad range of sectors. Additionally, it would be beneficial for both the public and private sectors to work together to create inclusive solutions that will reduce societal disparities and protect the environment at the same time.

As AI becomes increasingly integral to sustainability, it presents opportunities and challenges. A more sustainable market requires businesses to adopt AI to reduce costs; as McKinsey (2022), several companies have reported that AI forecasting engines reduce costs by 10%−15% and improve their competitive position by automating up to 50% of workforce management tasks. However, the role of policymakers and urban planners in creating the conditions for AI innovations to thrive responsibly and inclusively cannot be overstated. Integrating AI into sustainable practices requires balancing technological advancements with ethical considerations. AI can be a powerful force for sustainable development if stakeholders create a collaborative atmosphere, address barriers, and promote transparency. As a result, businesses, societies, and the environment will all benefit. By examining the intersection of AI and urban sustainability in a new manner, the article introduces a fresh perspective to the literature because its analysis is not comprehensively covered in the current literature. It is valuable to synthesize existing literature to highlight trends and develop a strong foundation for understanding AI's role in business.
